# Natural History and Clinical Outcomes of HBeAg-Negative Chronic Hepatitis B Patients in the “Grey Zone”: A Longitudinal Cohort Study

**DOI:** 10.3390/jcm15207832

**Published:** 2026-10-10

**Authors:** Yunling Xue, Mengyuan Liu, Guorui Wang, Qiao Tang, Jingsong Wang, Jing Zhang, Yi Zeng, Mingli Peng, Xiaoqing Liu, Peng Hu

**Affiliations:** The Key Laboratory of Molecular Biology for Infectious Diseases, Department of Infectious Diseases, Institute for Viral Hepatitis, Chinese Ministry of Education, The Second Affiliated Hospital of Chongqing Medical University, 76# Linjiang Road, Yuzhong District, Chongqing 400010, China

**Keywords:** chronic hepatitis B, Grey Zone, inactive carrier, disease progression

## Abstract

**Introduction and Objectives:** Up to one-third of treatment-naïve, HBeAg-negative chronic hepatitis B (CHB) patients with normal ALT do not meet the strict criteria for the inactive carrier (IC) phase, falling into a “Grey Zone” (GZIC). We aimed to delineate the longitudinal outcomes of GZIC patients compared with strictly defined IC patients. **Patients and Methods:** This retrospective cohort study included 386 HBeAg-negative, ALT-normal CHB patients (125 GZIC, 261 IC). Endpoints included phase transition to immune-active stages, antiviral initiation, and end-stage liver disease (cirrhosis or hepatocellular carcinoma (HCC)). Cumulative incidence was assessed using Kaplan–Meier analysis. **Results:** Over a median follow-up of 4.5 years, the GZIC cohort exhibited significantly higher cumulative incidence of phase transition (36.8% vs. 13.8%, *p* < 0.001), antiviral initiation (37.6% vs. 18.8%, *p* = 0.00014), and end-stage liver disease (4.8% vs. 1.5%, *p* = 0.049) compared with the IC cohort. All incident HCC cases occurred exclusively in the GZIC group. Multivariate analysis identified baseline GZIC status, male sex, and elevated AFP as independent risk factors. A lower ALT cutoff of 24.5 U/L was superior to 40 U/L in predicting phase transition (*p* < 0.01). Sex-stratified analyses confirmed that GZIC remained an independent risk factor for phase transition in both male and female patients (both *p* < 0.05). **Conclusions:** The GZIC phase is not a benign state. Compared with true inactive carriers, GZIC patients face a significantly elevated risk of progression and liver-related complications, supporting proactive surveillance and timely antiviral intervention.

## 1. Introduction

Chronic hepatitis B (CHB) virus infection remains a formidable global public health challenge, carrying a substantial risk for progressive liver disease, including fibrosis, cirrhosis, and hepatocellular carcinoma (HCC) [1,2,3,4]. The natural history of CHB is highly dynamic and complex, driven by the continuous interplay between viral replication and the host immune response. A comprehensive understanding of this natural course is imperative for optimizing clinical management, particularly regarding disease monitoring and the timing of antiviral therapy [5]. Based on the American Association for the Study of Liver Diseases (AASLD) 2018 guidance [4], the clinical course of CHB is conventionally categorized into four classical phases: immune-tolerant, HBeAg-positive immune-active, inactive CHB (IC), and HBeAg-negative immune-active (IA) phases [4].

Among these distinct phases, the IC state is highly prevalent, encompassing approximately 35% to 40% of treatment-naïve patients with CHB [6,7,8]. Accurate classification and rigorous longitudinal monitoring of this population are of significant clinical interest due to the inherent risk of disease reactivation. However, complicating this clinical paradigm is the observation that up to one-third of CHB patients present with discordant virological and biochemical profiles that fail to fulfill the strict criteria for any of the four classical phases. These individuals are increasingly recognized as residing in a clinical “Grey Zone” (GZ) [9,10,11,12,13]. Within this heterogeneous GZ population, a specific subset of HBeAg-negative patients with normal alanine aminotransferase (ALT) levels exhibit HBV DNA titers that deviate from the strict inactive criteria, placing them into a “Grey Zone inactive” (GZIC) category.

Currently, the natural history and long-term prognosis of patients within the GZIC cohort remain poorly characterized. The underlying clinical characteristics of these patients are complex, leaving their clinical significance, optimal follow-up strategies, and indications for therapeutic intervention largely undefined. Emerging evidence hypothesizes that the GZIC population may possess distinct disease trajectories compared with strictly defined IC patients. Therefore, the primary objective of this study was to systematically delineate and compare the longitudinal natural histories of true IC and GZIC cohorts. Specifically, we evaluated the rates of phase transition to the immune-active (IA) or Grey Zone immune-active (GZIA) stages, the incidence of antiviral therapy initiation, and the development of liver-related clinical endpoints.

## 2. Methods

### 2.1. Study Design and Patient Cohort

This was a longitudinal, retrospective cohort study enrolling patients with treatment-naïve chronic HBV infection (defined as HBsAg positivity for >6 months). All patients were HBeAg-negative with normal ALT levels at baseline and received clinical care at a tertiary teaching hospital between January 2011 and October 2023. The median follow-up duration for the entire cohort was 4.5 years (interquartile range [IQR], 2.4–6.8 years), with no statistically significant difference in follow-up time observed between the IC and GZIC groups (*p* = 0.5).

**Figure 1:**
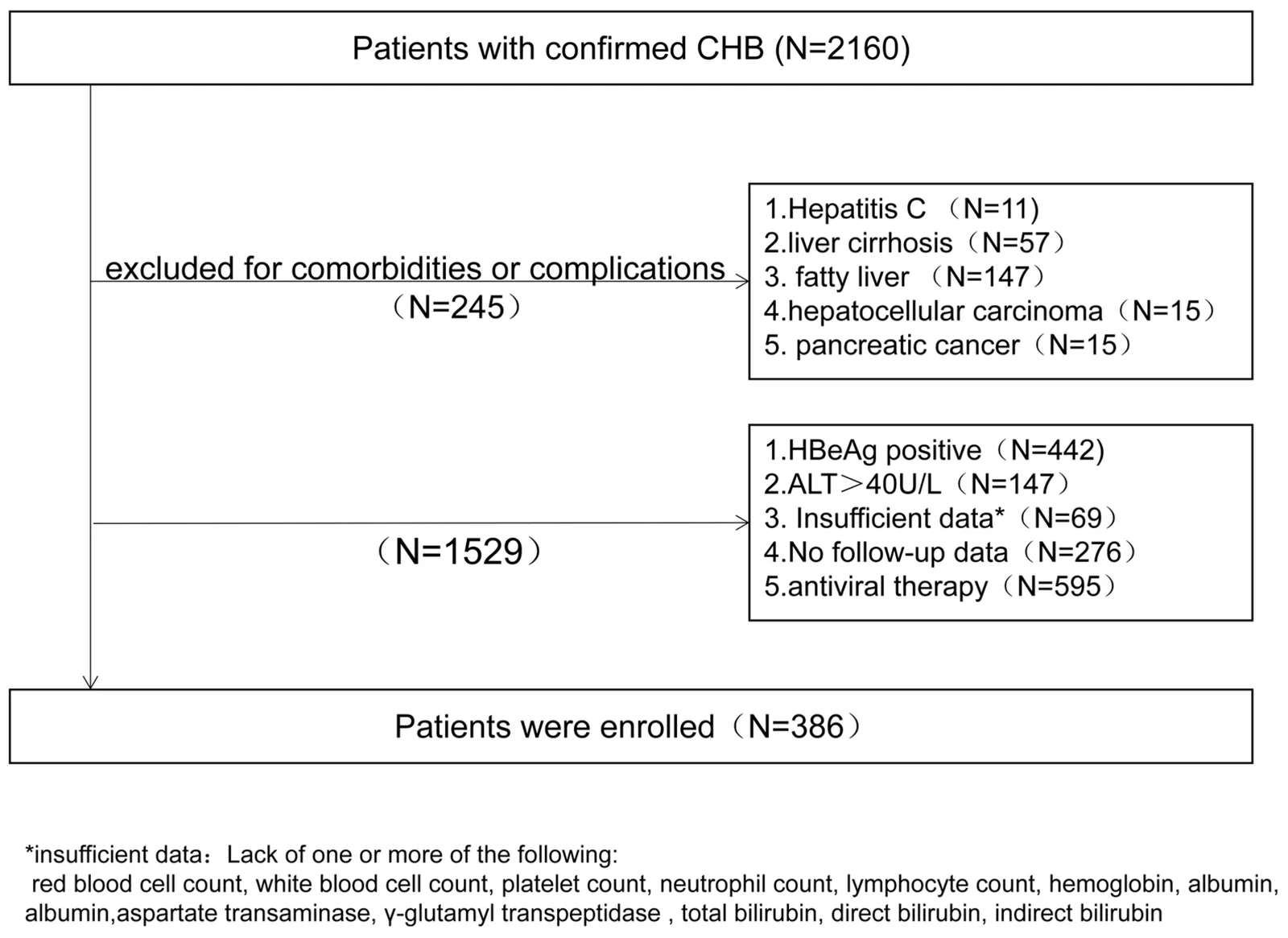
Flow diagram of patient selection.

As depicted in the patient selection flowchart (Figure 1), an initial cohort of 2160 CHB patients was screened. Ultimately, 386 patients were enrolled after stringent exclusions. A total of 245 patients were excluded due to competing comorbidities or baseline complications, including hepatitis C virus co-infection, hepatic steatosis, established cirrhosis, HCC, or pancreatic cancer. An additional 1529 patients were excluded for the following reasons: HBeAg positivity *(n* = 442), baseline ALT > 40 U/L (*n* = 147), incomplete baseline data (*n* = 69), loss to follow-up *(n* = 276), and ongoing antiviral therapy at baseline (*n* = 595). The study protocol was approved by the institutional review board of the participating hospital, conforming to the ethical guidelines of the Declaration of Helsinki.

### 2.2. Clinical and Laboratory Assessments

Demographic parameters (age, sex, family history of HBV) and clinical outcome data (initiation of antiviral therapy) were systematically extracted from electronic medical records. Baseline laboratory indices were utilized to stratify the immunological status of patients and assess longitudinal disease progression.

Serum ALT levels were quantified using an automated analyzer (Hitachi 7600-020, Tokyo, Japan), with the upper limit of normal (ULN) defined as 40 U/L. Serum HBV DNA viral loads were measured using the Roche Amplicor/COBAS TaqMan HBV test v2.0 (Roche Molecular Diagnostics, Pleasanton, CA, USA; lower limit of detection [LLD]: 100 IU/mL). Quantitative HBsAg testing was performed using the Roche COBAS HBsAg II-Q assay (LLD: 0.05 IU/mL). HBeAg status was determined utilizing the Abbott Architect system (Abbott GmbH & Co. KG, Wiesbaden, Germany; LLD: 0.59 PEIU/mL).

### 2.3. Diagnostic Criteria and Clinical Endpoints

The immunological phases of CHB were strictly defined in accordance with the AASLD 2018 Hepatitis B Guidance (Table S1) [4]. Although the term “inactive carrier” has been increasingly questioned and the most recent EASL guidelines propose the alternative designation “HBeAg-negative chronic HBV infection,” we retained the term “IC” in this study because our classification is based on the AASLD 2018 criteria and the term “IC” is widely used in the existing literature on the Grey Zone [4,14]. Patients failing to meet the rigorous criteria for inactive CHB but presenting with persistently normal ALT (≤40 U/L) were classified into the GZIC cohort. Specifically, GZIC was defined by either of the following criteria:

(a) ALT < 40 U/L, HBV DNA > 2000 IU/mL, with APRI < 1.5, FIB-4 < 3.25, and GPR < 0.32; or

(b) ALT < 40 U/L, regardless of HBV DNA level (≤2000 IU/mL or >2000 IU/mL), but fulfilling one or more of the following non-invasive fibrosis criteria: APRI ≥ 1.5, FIB-4 ≥ 3.25, or GPR ≥ 0.32 [15,16,17]. Non-invasive serological markers were calculated to estimate liver fibrosis severity: [16,18]


APRI = ([AST/ULN] × 100)/Platelet count (10^9^/L)

FIB-4 = age ([yr] × AST [U/L])/((PLT [10^9^/L]) × (ALT [U/L])^1/2^)

GPR = ([GGT/ULN] × 100)/Platelet count (10^9^/L).

The primary clinical endpoints evaluated in this study were: (1) immunological phase transition to IA or GZIA (HBeAg-negative); (2) the initiation of guideline-indicated antiviral therapy; and (3) the development of end-stage liver disease events (defined as the incident diagnosis of cirrhosis or HCC). Diagnoses of cirrhosis and HCC were established based on AASLD guidelines, utilizing a combination of imaging modalities (ultrasound, contrast-enhanced magnetic resonance imaging [MRI]), transient elastography (FibroScan), and tumor biomarkers (AFP, PIVKA-II) [19,20].

### 2.4. Statistical Analysis

Continuous variables were expressed as mean ± standard deviation (SD) or median (interquartile range [IQR]) depending on data distribution, and between-group differences were evaluated using the Student’s *t*-test or Mann–Whitney U test, as appropriate. Categorical variables were presented as frequencies and percentages, and comparisons were performed using the Chi-square test or Fisher’s exact test. Cumulative incidences of outcome events were estimated via the Kaplan–Meier method, with differences between strata assessed by the log-rank test.

Univariate and multivariate Cox proportional hazards regression models were constructed to identify independent risk factors for disease progression. Variables with a *p*-value < 0.05 in the univariate analysis were advanced to the multivariate model. To avoid collinearity among fibrosis-related markers, only APRI was retained when APRI, GPR, and FIB-4 were simultaneously significant in univariate analysis.

All statistical analyses were conducted using R software (version 4.0, R Foundation for Statistical Computing, Vienna, Austria). A two-sided *p*-value < 0.05 was considered statistically significant.

### 2.5. Ethical Statements

This retrospective study was conducted using de-identified medical records from patients treated at the Second Affiliated Hospital of Chongqing Medical University. The study protocol was reviewed and approved by the Ethics Committee of the Second Affiliated Hospital of Chongqing Medical University (Approval No. 2023[36]). Owing to the retrospective nature of the study and the use of anonymized data, the requirement for individual informed consent was waived by the Ethics Committee. All procedures performed were in accordance with the ethical standards of the institutional research committee and with the 1964 Helsinki Declaration and its later amendments or comparable ethical standards.

## 3. Results

### 3.1. Baseline Demographic and Clinical Characteristics

Following rigorous selection criteria, 386 patients were included in the final analysis. At baseline, 261 patients (67.6%) met the strict criteria for the inactive CHB (IC) phase, whereas 125 patients (32.4%) were classified into the GZIC phase. The baseline demographic and clinical profiles of the cohort are detailed in Table 1. Notably, male predominance was significantly more pronounced in the GZIC cohort compared with the IC cohort (64.8% vs. 43.7%, *p* < 0.001). Furthermore, significant between-group differences were observed across a spectrum of biochemical and hematological indices, including ALT, AST, γ-GT, platelets (PLT), hemoglobin (HB), APRI, GPR, platelet-to-lymphocyte ratio (PLR), and AFP (all *p* < 0.05; Table 1).

### 3.2. Elevated Risk of Phase Transition to IA or GZIA in the GZIC Cohort

**Figure 2:**
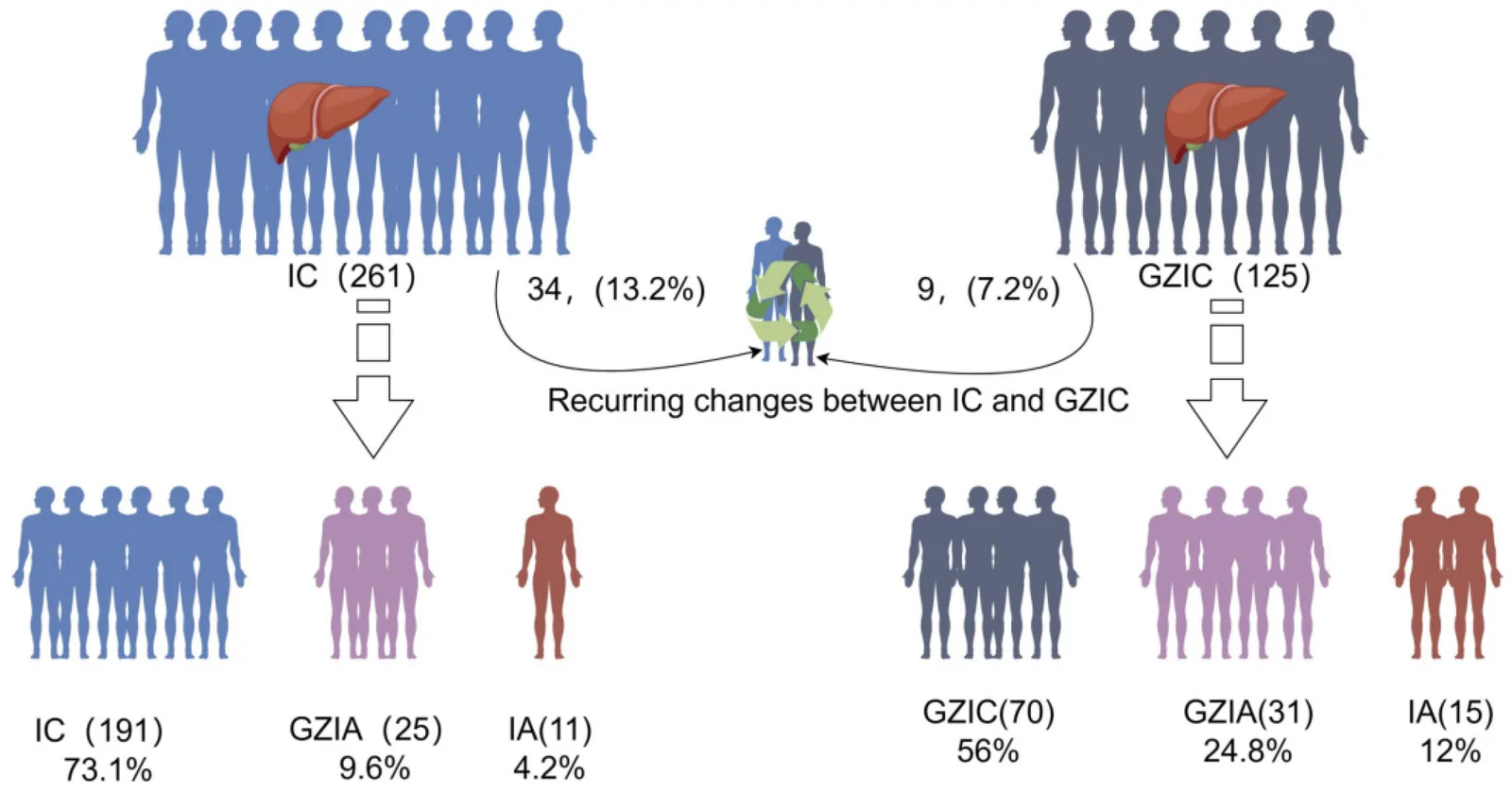
Changes in the immune states of patients.

**Figure 3:**
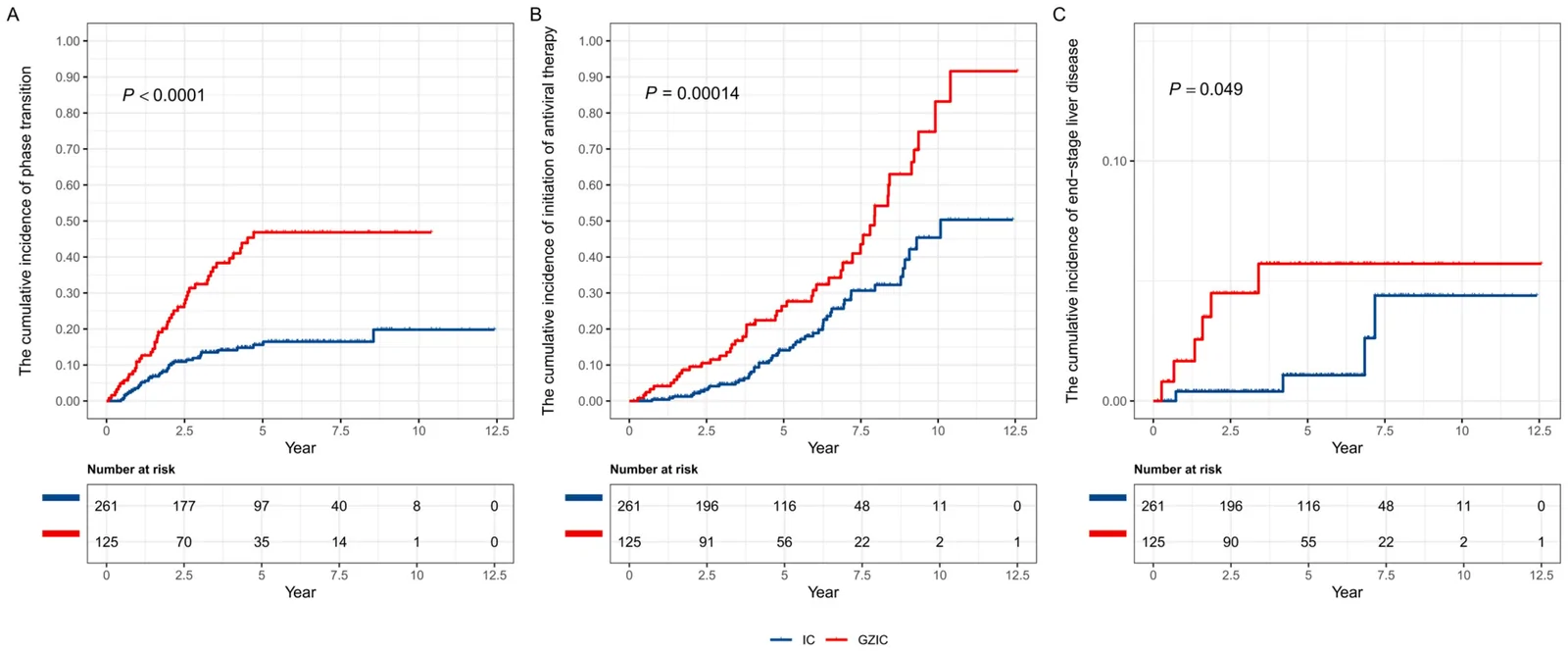
Cumulative event rates of outcomes. (**A**) Cumulative incidence of phase transition to IA or GZIA (IC vs. GZIC); (**B**) cumulative incidence of antiviral therapy initiation (IC vs. GZIC); (**C**) Cumulative incidence of end-stage liver disease (IC vs. GZIC).

During the follow-up period, 82 patients experienced a phase transition to IA or GZIA. Baseline immune status was persistent in 191 IC patients (73.1%) and 70 GZIC patients (56.0%). Fluctuation between the IC and GZIC states occurred in 34 IC patients (13.1%) and 9 GZIC patients (7.2%). Crucially, the rates of progression toward more active disease phases were markedly higher in the GZIC group. Transition to GZIA or IA occurred in 24.8% (31/125) and 12.0% (15/125) of GZIC patients, respectively, compared with only 9.6% (25/261) and 4.2% (11/261) of IC patients (Figure 2). Kaplan–Meier survival analysis confirmed that the cumulative incidence of phase transition (to IA or GZIA) was significantly higher in the GZIC cohort than in the IC cohort (*p* < 0.001; Figure 3A).

Multivariate Cox proportional hazards regression revealed that baseline GZIC status, male sex, and elevated baseline AFP were independent risk factors for immunological progression to IA/GZIA (all *p* < 0.05; Table 2). Higher hemoglobin levels were also associated with lower risk in the multivariate model, but this association was attenuated after adjustment for sex alone (*p* = 0.139), suggesting possible confounding by sex. When analyzed for transition to the IA stage alone, GZIC remained a robust independent risk factor (*p* = 0.008; Table S2).

Given the ongoing clinical debate regarding the optimal ULN for ALT and its profound link to host immune status, we performed a receiver operating characteristic (ROC) curve analysis for baseline ALT. The optimal ALT cutoff value for predicting phase transition was identified as 24.5 U/L (AUC = 0.687, 95% CI: 0.619–0.755) (Figure S1A). When stratifying the cohort using this newly identified threshold, patients with high-normal ALT (24.5–40 U/L) exhibited significantly higher cumulative incidence rates for transition to GZIA/IA (*p* < 0.001) and to IA alone (*p* = 0.0026) compared with those with strictly low-normal ALT (<24.5 U/L) (Figure S1B,C).

### 3.3. Increased Initiation of Antiviral Therapy in GZIC Patients

Over the follow-up period, antiviral therapy was initiated in 96 of the 386 patients based on evolving clinical indications. The proportion of patients requiring therapeutic intervention was approximately twice as high in the GZIC cohort (47/125, 37.6%) as in the IC cohort (49/261, 18.8%). Kaplan–Meier analysis demonstrated a significantly higher cumulative incidence of antiviral therapy initiation in the GZIC group (*p* = 0.00014; Figure 3B). Further multivariate Cox regression analysis identified GZIC status, APRI score, and AFP levels as independent predictors for the subsequent need to initiate antiviral therapy (all *p* < 0.05; Table S3).

### 3.4. Higher Incidence of End-Stage Liver Disease in the GZIC Cohort

A total of ten incident cases of end-stage liver disease (comprising 2 cases of HCC and 8 cases of cirrhosis) were recorded during follow-up. Among these, the absolute incidence rate was disproportionately higher in the GZIC group (6/125, 4.8%) compared with the IC group (4/261, 1.5%). Strikingly, both incident cases of HCC occurred exclusively within the GZIC cohort. Kaplan–Meier analysis confirmed that GZIC patients harbored a significantly higher cumulative risk of developing end-stage liver-related events compared with their strictly inactive counterparts (*p* = 0.049, Figure 3C).

### 3.5. Sex-Stratified Outcomes

Given the male predominance in the GZIC cohort, we further stratified the analyses by sex (Tables S4 and S5). Both male and female GZIC patients had significantly higher ALT, Γ-GT, APRI, and GPR levels compared with their IC counterparts (all *p* < 0.05). Male GZIC patients additionally had significantly lower PLT and PLR than male IC patients, whereas female GZIC patients had significantly higher AFP levels than female IC patients.

In sex-stratified Cox regression, GZIC remained an independent risk factor for phase transition in both male (*p* = 0.015) and female patients (*p* < 0.001). For antiviral therapy initiation, the association was significant in female patients (*p* = 0.0005) but not in male patients (*p* = 0.640). For end-stage liver disease, the number of events was too small for sex-stratified Cox regression (male: 6; female: 4); descriptively, the absolute incidence was highest in male GZIC patients (5/81, 6.2%).

## 4. Discussion

The accurate stratification of immune status in chronic hepatitis B (CHB) is paramount for predicting long-term prognosis and optimizing the timing of antiviral therapy [21]. In this study, we specifically focused on patients traditionally categorized under the inactive CHB (IC) umbrella and those residing in its corresponding clinical Grey Zone (GZIC) [6,8]. Our cohort revealed that nearly one-third (32.4%) of treatment-naïve patients with normal ALT and HBeAg negativity fell into the GZIC category. This proportion is highly consistent with previous epidemiological studies (30–40%), emphasizing that the Grey Zone represents a substantial and non-negligible clinical population [4,6,22,23]. While the prognosis of strictly defined IC patients is generally considered benign [1,2,4], our findings strongly suggest that the GZIC population possesses a distinct, heterogeneous, and considerably more progressive natural history.

Longitudinal tracking over a median of 4.5 years robustly demonstrated that patients in the GZIC phase experienced significantly higher cumulative rates of disease progression across all evaluated endpoints compared with their IC counterparts. Specifically, GZIC patients were more than twice as likely to transition to the immune-active (IA/GZIA) phases and were twice as likely to require the initiation of antiviral therapy. Most alarmingly, incident cases of end-stage liver disease, particularly HCC, occurred disproportionately within the GZIC cohort. Multivariate Cox regression further consolidated baseline GZIC status, male sex, and elevated AFP as robust independent predictors of immunological progression and the need for therapeutic intervention. Elevated AFP may reflect subclinical hepatocellular injury and regeneration driven by persistent HBV cccDNA and viral integration, consistent with its role as a marker of “occult active disease” in patients with normal ALT [24,25]. In contrast, the protective association of higher hemoglobin was attenuated after adjustment for sex (*p* = 0.139), suggesting confounding by sex rather than a true biological effect.

The long-term prognosis of grey-zone patients has been a subject of considerable debate in recent literature. Our conclusions diverge from the findings of Bonaci et al. and Oliveri et al., who reported a benign prognosis for HBeAg-negative patients in the Grey Zone, suggesting that antiviral therapy could be safely delayed [10,12]. This discrepancy is likely multifactorial. First, the populations in the aforementioned studies were predominantly Caucasian, with HBV genotypes A and D being the most prevalent. In contrast, our cohort consisted entirely of Chinese patients, a population in which genotypes B and C predominate. It is well-documented that genotypes B and C carry a higher risk of chronicity, necroinflammatory exacerbations, and greater oncogenic potential compared with genotypes A and D [26]. Second, the definition of “active hypoviremia” used by Oliveri et al. (HBV DNA consistently ≤ 20,000 IU/mL) represents only a sub-fraction of the broader GZIC criteria utilized in our study, where patients with higher viremia were also included [11]. Given that HBV DNA replication is a core driver of disease progression, this definitional variance likely contributes to the divergent clinical outcomes observed [27,28].

Conversely, our findings are heavily corroborated by a large-scale, multicenter cohort study of 3366 patients from the United States and Asia. That study demonstrated that patients with persistent indeterminate (Grey Zone) staging had a 14-fold higher risk of HCC than strictly inactive carriers [7,29]. Furthermore, multiple recent investigations from Asian centers have consistently confirmed that without antiviral intervention, grey-zone patients face a substantially elevated risk of disease progression [30,31]. Together with our data, this growing body of evidence unequivocally suggests that GZIC is a high-risk state, especially within Asian populations, warranting rigorous monitoring and proactive antiviral management to avert severe liver-related events [4,32].

Beyond baseline immune stratification, our study identified male sex as an independent risk factor for adverse clinical events, aligning with established evidence that male patients with CHB generally exhibit a more aggressive disease course [33,34,35]. The mechanisms may include hormonal factors (androgens promote HBV replication and hepatocarcinogenesis, whereas estrogens are protective), sex-based immune differences, behavioral and metabolic factors, and androgen receptor–mediated enhancement of viral transcription [36,37,38]. In our cohort, male sex was an independent risk factor for phase transition, and male GZIC patients had more severe fibrosis markers and more end-stage liver disease events than female GZIC patients. In sex-stratified analyses, GZIC remained an independent risk factor for phase transition in both male and female patients. For antiviral therapy initiation, the association was significant in female patients but not in male patients. As this endpoint reflects clinical decision-making in addition to disease progression, this finding suggests that male GZIC patients may be undertreated despite their higher risk. For end-stage liver disease, the number of events was too small for sex-stratified Cox regression; descriptively, the absolute incidence was highest in male GZIC patients. Given that antiviral therapy has been shown to reduce HCC risk by 70% among patients with indeterminate-phase CHB, a more proactive treatment strategy should be considered for GZIC patients, particularly male patients or those with additional risk factors [39].

More importantly, our post-hoc analysis of ALT thresholds adds significant weight to the ongoing clinical debate regarding the optimal upper limit of normal (ULN) for ALT. We identified an optimal ALT cutoff of 24.5 U/L for predicting phase transition, which aligns perfectly with a nationwide histological study of 1391 CHB patients demonstrating that an ALT threshold of 25 U/L best detects significant liver necroinflammation [22]. Consistent with reports by Duan et al. and others [35,40], our survival analysis confirmed that patients with high-normal ALT (24.5–40 U/L) experienced significantly higher rates of disease progression than those with strictly low-normal ALT. Recent studies have proposed combining ALT thresholds with age (e.g., a 30-year cut-off) to improve the accuracy of assessing liver status in GZ patients [41]. These findings strongly suggest that the current uniform ALT threshold of 40 U/L—applied regardless of sex or age—fails to identify a substantial proportion of patients at risk of progression, whereas adjusting the ALT decision threshold could more sensitively identify candidates for antiviral therapy and benefit a broader patient population [14,42].

The GZIC population, however, is not a uniform entity. Recent studies have classified indeterminate CHB into 10 types [43]. In our research the heterogeneity that drives progression toward end-stage liver disease likely arises from three interrelated domains. First, viral factors: our GZIC definition includes patients with HBV DNA both ≤2000 and >2000 IU/mL, and recent studies suggest that HBV DNA > 2000 IU/mL identifies a subgroup with substantially higher progression risk [44]. Quantitative HBsAg and HBcrAg may further refine this stratification. Second, host factors: age, sex, high-normal ALT, and metabolic comorbidities all contribute to heterogeneous outcomes within GZIC. In our sex-stratified analysis, male GZIC patients had more severe baseline fibrosis markers and more end-stage liver disease events than female GZIC patients. Third, histological factors: In our cohort, GZIC patients were enrolled partly based on elevated APRI/FIB-4/GPR, which likely enriched for underlying histological disease. However, non-invasive markers cannot fully replace liver biopsy for assessing necroinflammation and fibrosis. Recent studies have reported that >70% of GZ patients have significant histological disease despite normal ALT [45]. Therefore, liver biopsy or non-invasive assessment should be considered in GZIC patients to guide treatment decisions, and future studies incorporating HBcrAg, quantitative HBsAg, and liver histology are needed to refine risk stratification.

Several limitations of this study must be acknowledged. First, the retrospective, single-center design may introduce inherent selection biases. Second, the categorization of immune status was based on single-timepoint baseline assessments; dynamic fluctuations in viral and biochemical markers prior to enrollment could not be fully captured, potentially leading to misclassification bias. Third, the evaluation of liver fibrosis relied on non-invasive serological scoring systems (APRI, FIB-4, GPR) rather than the gold standard of liver biopsy. Fourth, the end-stage liver disease analysis was limited by the small number of events (*n* = 10), and the sex-stratified analysis for this endpoint should be interpreted as exploratory. Future prospective, multicenter studies incorporating histological assessments are needed to validate our findings.

## 5. Conclusions

In summary, this study highlights that over one-third of HBeAg-negative, ALT-normal CHB patients reside in the GZIC phase. Rather than representing a benign state, GZIC is characterized by a significantly higher risk of transition to immune-active phases, a greater need for antiviral therapy, and an increased incidence of end-stage liver disease compared with strictly inactive CHB. These findings challenge the traditional paradigm of “watchful waiting” for grey-zone patients and advocate for closer virological surveillance and the timely initiation of antiviral therapy to optimize long-term clinical outcomes. However, these findings are derived from an Asian cohort, and validation in Western populations is needed.

## Figures and Tables

**Table 1:** Baseline table for study population.

	IC	GZIC	*p*
*n*	261	125	
family history			0.335
YES (%)	26 (10.00)	8 (6.40)	
NO (%)	235 (90.00)	117 (93.60)	
sex			<0.001
male (%)	114 (43.70)	81 (64.80)	
Female (%)	147 (56.30)	44 (35.20)	
age (median (IQR))	40.45 (31.99, 46.35)	41.57 (34.57, 47.00)	0.283
A (median (IQR))	45.90 (44.60, 47.40)	46.10 (44.10, 47.60)	0.86
ALT (median (IQR))	19.00 (15.00, 25.00)	26.00 (18.00, 33.00)	<0.001
AST (median (IQR))	22.00 (19.00, 26.00)	25.00 (22.00, 29.00)	<0.001
Γ-GT (median (IQR))	16.00 (13.00, 19.00)	26.00 (17.00, 34.00)	<0.001
TB (median (IQR))	11.80 (9.50, 15.22)	12.40 (10.00, 16.10)	0.569
DB (median (IQR))	4.60 (3.60, 5.80)	4.60 (3.40, 6.35)	0.942
IDB (median (IQR))	7.50 (5.60, 9.80)	7.20 (5.30, 10.10)	0.942
APRI (median (IQR))	0.29 (0.23, 0.38)	0.36 (0.29, 0.49)	<0.001
GPR (median (IQR))	0.17 (0.13, 0.21)	0.33 (0.20, 0.45)	<0.001
FIB4 (median (IQR))	0.48 (0.33, 0.67)	0.49 (0.32, 0.72)	0.425
NLR (median (IQR))	1.90 (1.47, 2.47)	1.89 (1.36, 2.45)	0.551
PLR (median (IQR))	108.47 (88.48, 132.60)	101.85 (78.79, 120.61)	0.001
RBC (median (IQR))	4.73 (4.38, 5.08)	4.89 (4.51, 5.25)	0.051
HB (median (IQR))	143.00 (132.00, 155.00)	151.00 (136.00, 160.00)	0.004
WBC (median (IQR))	5.59 (4.85, 6.44)	5.63 (4.78, 6.50)	0.971
N (median (IQR))	3.29 (2.69, 3.99)	3.28 (2.71, 4.17)	0.985
L (median (IQR))	1.71 (1.45, 2.04)	1.76 (1.45, 2.12)	0.544
PLT (mean (SD))	193.70 (47.14)	170.24 (50.22)	<0.001
AFP (median (IQR))	2.87 (2.06, 4.06)	3.44 (2.75, 4.77)	0.001

**Table 2:** Cox regression analysis of factors associated with conversion to IA or GZIA.

Characteristics	Univariate Analysis		Multivariate Analysis	
	HR (95% CI for HR)	*p* Value	HR (95% CI for HR)	*p* Value
Group		<0.001		0.005
GZIC	Reference		Reference	
IC	0.32 (0.20–0.49)		0.44 (0.25–0.78)	
family		0.502		
NO	Reference			
YES	0.75 (0.33–1.73)			
sex		<0.001		0.004
female	Reference		Reference	
male	2.92 (1.81–4.74)		3.60 (1.50–8.64)	
age	1.011 (0.989–1.033)	0.335		
A	1.066 (0.975–1.166)	0.160		
ALT	1.071 (1.044–1.099)	<0.001	1.029 (0.995–1.063)	0.092
AST	1.021 (1.009–1.034)	<0.001	1.025 (0.977–1.076)	0.316
Γ-GT	1.009 (1.004–1.014)	0.001	1.011 (0.998–1.024)	0.100
TB	1.002 (0.970–1.034)	0.924		
DB	1.037 (0.940–1.145)	0.468		
IDB	0.999 (0.959–1.041)	0.973		
GPR	2.395 (1.693–3.387)	<0.001		
FIB4	0.827 (0.519–1.317)	0.423		
RBC	2.338 (1.488–3.672)	<0.001	1.645 (0.834–3.242)	0.151
WBC	0.998 (0.845–1.180)	0.982		
N	0.954 (0.779–1.170)	0.653		
L	1.062 (0.691–1.630)	0.785		
HB	1.018 (1.004–1.032)	0.011	0.965 (0.946–0.986)	<0.001
PLT	1.000 (0.996–1.005)	0.865		
NLR	0.961 (0.744–1.242)	0.761		
PLR	1.000 (0.994–1.006)	0.953		
AFP	1.009 (1.002–1.017)	0.012	1.012 (1.004–1.020)	0.002

## Data Availability

The original contributions presented in this study are included in the article/Supplementary Materials. Further inquiries can be directed to the corresponding authors.

## Supplementary Materials

The following supporting information can be downloaded at: https://www.mdpi.com/article/10.3390/jcm15207832/s1, Figure S1: (A) ROC curves for the outcome event of ALT predicting conversion to IA or GZIA; (B) cumulative event rates comparing conversion to IA or GZIA after subgrouping with the optimal cut-off of 25 for ALT; (C) cumulative event rates comparing conversion to IA after subgrouping with the optimal cut-off of 25 for ALT; Table S1: Definitions of immune states of CHB; Table S2: Cox regression analysis of factors associated with conversion to IA; Table S3: Cox regression analysis of factors associated with Antiviral therapy; Table S4: Baseline characteristics stratified by sex and status (IC vs. GZIC); Table S5: Sex-stratified Cox regression analysis of factors associated with clinical outcomes.
