# Supplementary material for: Natural History and Clinical Outcomes of HBeAg-Negative Chronic Hepatitis B Patients in the “Grey Zone”: A Longitudinal Cohort Study
Source: J Clin Med. 2026 Oct 10;15(20):7832. doi: 10.3390/jcm15207832 (PMC13705509; doi:10.3390/jcm15207832)
Supplement: Supplementary file 1 [file jcm-15-07832-s001.zip › Table S1.pdf]

Table S1. Definitions of immune states of CHB

	HBeAg	ALT	DNA	APRI/GPR/FIB-4
IC	-	＜ 40	DNA ＜ 2000IU/mL	normal‡
GZIC	-	＜ 40	DNA ≥ 2000IU/mL	normal
			DNA ＜ 2000IU/mL	elevated*
IA	-	≥ 80	DNA ≥ 2000IU/mL	normal/elevated
GZIA	-	40 ≤ ALT ＜ 80	DNA＜2000IU/mL or DNA ≥ 2000IU/mL	normal/elevated
		≥ 80	DNA ＜ 2000IU/mL	normal/elevated

‡The upper limit of normal of APRI/FIB-4/GPR: APRI ＜ 1.5，GPR ＜ 0.32，FIB-4 ＜ 3.25.

*elevated：Any one or more of APRI, FIB4, GPR is higher than normal.
