# Supplementary material for: Natural History and Clinical Outcomes of HBeAg-Negative Chronic Hepatitis B Patients in the “Grey Zone”: A Longitudinal Cohort Study
Source: J Clin Med. 2026 Oct 10;15(20):7832. doi: 10.3390/jcm15207832 (PMC13705509; doi:10.3390/jcm15207832)
Supplement: Supplementary file 1 [file jcm-15-07832-s001.zip › Table S2.pdf]

Table S2. Cox regression analysis of factors associated with conversion to IA.

Characteristics	Univariate analysis		Multivariate analysis	
	HR (95% CI for HR)	p.value	HR (95% CI for HR)	p.value
Group				
GZIC	Reference		Reference	
IC	0.3 (0.138-0.655)	0.002	0.348（0.156，0.784）	0.01
family				
NO	Reference			
YES	0.8 (0.188-3.38)	0.758		
age	1 (0.982-1.06)	0.296		
sex				
female	Reference			
male	2.1 (0.944-4.76)	0.069		
ALT	1.04 (1.01-1.1)	0.038	1.029（0.984，1.077）	0.201
A	1 (0.882-1.18)	0.789		
AST	1 (0.998-1.04)	0.081		
Γ-GT	1 (0.997-1.02)	0.141		
TB	0.96 (0.888-1.04)	0.318		
DB	0.96 (0.794-1.17)	0.693		
IDB	0.95 (0.852-1.05)	0.298		
APRI	0.91 (0.156-5.3)	0.915		
GPR	1 (0.55-1.9)	0.946		
FIB4	0.85 (0.384-1.9)	0.699		
RBC	1.8 (0.825-3.95)	0.139		
WBC	0.96 (0.716-1.3)	0.803		
N	0.84 (0.575-1.24)	0.384		
L	1 (0.782-1.28)	0.993		
HB	1 (0.984-1.03)	0.553		
PLT	1 (0.997-1.01)	0.204		
NLR	0.77 (0.461-1.28)	0.315		
PLR	1 (0.988-1.01)	0.884		
AFP	1 (0.981-1.03)	0.792		
