# Supplementary material for: Natural History and Clinical Outcomes of HBeAg-Negative Chronic Hepatitis B Patients in the “Grey Zone”: A Longitudinal Cohort Study
Source: J Clin Med. 2026 Oct 10;15(20):7832. doi: 10.3390/jcm15207832 (PMC13705509; doi:10.3390/jcm15207832)
Supplement: Supplementary file 1 [file jcm-15-07832-s001.zip › Table S3.pdf]

Table S3. Cox regression analysis of factors associated with Antiviral therapy.

Characteristics	Univariate analysis		Multivariate analysis	
	HR (95% CI)	P	HR (95% CI)	P
**Group**		**<0.001**		**0.003**
GZIC	Reference		Reference	
IC	0.47 (0.31–0.70)		0.49 (0.30–0.79)	
**sex**		0.441		
female	Reference			
male	1.17 (0.78–1.75)			
**family**		0.575		
NO	Reference			
YES	1.20 (0.64–2.25)			
age	1.019 (0.998–1.039)	0.071		
A	0.966 (0.920–1.014)	0.165		
ALT	1.002 (0.980–1.025)	0.874		
**AST**	**1.028 (1.015–1.041)**	**<0.001**	0.955 (0.910–1.002)	0.059
Γ-GT	1.002 (0.994–1.010)	0.550		
TB	0.996 (0.963–1.031)	0.836		
DB	0.989 (0.895–1.092)	0.822		
IDB	0.990 (0.946–1.036)	0.670		
**APRI**	**5.512 (2.852–10.654)**	**<0.001**	**10.244 (2.143–48.974)**	**0.004**
GPR	1.883 (1.205–2.942)	0.006		

Characteristics	Univariate analysis		Multivariate analysis	
FIB4	1.209 (0.993–1.472)	0.059		
NLR	1.124 (0.903–1.399)	0.294		
PLR	0.999 (0.993–1.005)	0.681		
RBC	0.855 (0.571–1.279)	0.445		
HB	1.007 (0.994–1.020)	0.281		
WBC	0.948 (0.810–1.110)	0.510		
N	0.990 (0.825–1.186)	0.910		
L	0.719 (0.465–1.111)	0.138		
PLT	0.996 (0.991–1.000)	0.037		
**AFP**	**1.013 (1.006–1.021)**	**<0.001**	**1.012 (1.005–1.020)**	**0.002**

Table S3. Univariate and multivariate Cox regression analysis of factors associated with antiviral therapy initiation.

HR, hazard ratio; CI, confidence interval. Variables with $P<0.05$ in univariate analysis were entered into the multivariate model. APRI, GPR, and PLT were highly correlated (VIF > 5 when entered together); therefore, only APRI was retained in the multivariate model to avoid collinearity. HR for AFP is per 1 ng/mL increase.
