# Supplementary material for: Natural History and Clinical Outcomes of HBeAg-Negative Chronic Hepatitis B Patients in the “Grey Zone”: A Longitudinal Cohort Study
Source: J Clin Med. 2026 Oct 10;15(20):7832. doi: 10.3390/jcm15207832 (PMC13705509; doi:10.3390/jcm15207832)
Supplement: Supplementary file 1 [file jcm-15-07832-s001.zip › Table S4.pdf]

Table S4. Baseline characteristics stratified by sex and status (IC vs. GZIC).

Characteristics	Male IC (n=114)	Male GZIC (n=81)	P	Female IC (n=147)	Female GZIC (n=44)	P
Status (%)			<0.001			<0.001
IC	114 (100.0)	0 (0.0)		147 (100.0)	0 (0.0)	
GZIC	0 (0.0)	81 (100.0)		0 (0.0)	44 (100.0)	
family history (%)			0.091			0.716
No	103 (90.4)	79 (97.5)		132 (89.8)	38 (86.4)	
Yes	11 (9.6)	2 (2.5)		15 (10.2)	6 (13.6)	
age (median [IQR])	40.13 [30.27, 45.97]	41.60 [34.95, 45.48]	0.235	41.34 [34.10, 46.38]	40.76 [30.02, 49.27]	0.626
A (median [IQR])	46.90 [45.50, 47.90]	46.60 [45.00, 48.30]	0.718	45.40 [43.90, 46.85]	45.30 [43.27, 46.47]	0.298
ALT (median [IQR])	23.00 [17.00, 28.75]	29.00 [24.00, 35.00]	<0.001	17.00 [14.00, 22.00]	22.50 [16.75, 27.00]	0.001
AST (median [IQR])	24.00 [21.00, 27.00]	25.00 [22.00, 29.00]	0.111	21.00 [18.00, 25.00]	24.50 [21.00, 29.50]	<0.001
Γ-GT (median [IQR])	17.00 [15.00, 22.00]	30.00 [24.00, 38.00]	<0.001	14.00 [12.00, 17.00]	16.00 [12.75, 19.25]	0.019
TB (median [IQR])	13.20 [10.40, 16.00]	13.15 [10.95, 16.55]	0.670	11.30 [9.00, 14.55]	10.80 [8.43, 13.90]	0.504

Characteristics	Male IC (n=114)	Male GZIC (n=81)	P	Female IC (n=147)	Female GZIC (n=44)	P
DB (median [IQR])	4.80 [4.10, 6.30]	5.00 [3.88, 6.62]	0.747	4.40 [3.40, 5.50]	3.60 [2.90, 5.23]	0.155
IDB (median [IQR])	8.00 [5.90, 10.00]	7.75 [5.97, 10.35]	0.994	6.90 [5.55, 9.35]	6.95 [5.10, 9.12]	0.820
APRI (median [IQR])	0.32 [0.25, 0.39]	0.36 [0.30, 0.48]	<0.001	0.27 [0.22, 0.36]	0.34 [0.26, 0.61]	0.001
GPR (median [IQR])	0.19 [0.16, 0.25]	0.37 [0.31, 0.49]	<0.001	0.15 [0.12, 0.20]	0.17 [0.13, 0.27]	0.008
FIB4 (median [IQR])	0.43 [0.30, 0.56]	0.46 [0.30, 0.70]	0.275	0.51 [0.36, 0.81]	0.59 [0.40, 0.90]	0.205
NLR (median [IQR])	1.88 [1.43, 2.37]	1.81 [1.33, 2.37]	0.557	1.96 [1.52, 2.50]	2.12 [1.56, 2.63]	0.599
PLR (median [IQR])	108.03 [84.33, 127.51]	94.64 [75.71, 115.17]	0.006	109.62 [89.76, 141.92]	108.89 [87.57, 121.83]	0.228
RBC (median [IQR])	5.10 [4.82, 5.33]	5.14 [4.89, 5.37]	0.749	4.45 [4.24, 4.72]	4.38 [4.14, 4.54]	0.107
HB (median [IQR])	157.00 [151.25, 163.00]	157.00 [150.00, 162.00]	0.973	133.00 [127.00, 141.00]	134.50 [128.00, 139.00]	0.858
WBC (median [IQR])	5.80 [5.25, 6.59]	5.76 [4.80, 6.52]	0.348	5.51 [4.64, 6.20]	5.58 [4.74, 6.40]	0.452
N (median [IQR])	3.33 [2.88, 3.98]	3.17 [2.65, 3.88]	0.215	3.25 [2.57, 4.04]	3.46 [3.01, 4.21]	0.286
L (median [IQR])	1.80 [1.52, 2.16]	1.81 [1.46, 2.20]	0.818	1.66 [1.38, 1.98]	1.69 [1.33, 1.90]	0.833

Characteristics	Male IC (n=114)	Male GZIC (n=81)	P	Female IC (n=147)	Female GZIC (n=44)	P
PLT (mean (SD))	193.89 (41.24)	167.64 (46.50)	<0.001	193.55 (51.39)	179.86 (50.54)	0.121
AFP (median [IQR])	3.08 [2.08, 4.19]	3.38 [2.86, 4.81]	0.082	2.71 [2.06, 3.86]	3.56 [2.56, 4.52]	0.019
