# Supplementary material for: Natural History and Clinical Outcomes of HBeAg-Negative Chronic Hepatitis B Patients in the “Grey Zone”: A Longitudinal Cohort Study
Source: J Clin Med. 2026 Oct 10;15(20):7832. doi: 10.3390/jcm15207832 (PMC13705509; doi:10.3390/jcm15207832)
Supplement: Supplementary file 1 [file jcm-15-07832-s001.zip › Table S5.pdf]

Table S5. Sex-stratified Cox regression analysis of factors associated with clinical outcomes

A. Phase transition

Characteristics	Male: Univariate		Male: Multivariate		Female: Univariate		Female: Multivariate	
	HR (95% CI)	P	HR (95% CI)	P	HR (95% CI)	P	HR (95% CI)	P
**Group**		**0.001**		**0.015**		**0.002**		**<0.001**
GZIC	Reference		Reference		Reference		Reference	
IC	0.43 (0.25–0.72)		0.46 (0.25–0.86)		0.27 (0.12–0.62)		0.22 (0.09–0.51)	
**family**		0.678				0.916		
NO	Reference				Reference			
YES	0.78 (0.25–2.50)				1.07 (0.32–3.60)			
age	1.004 (0.978–1.029)	0.784			1.042 (1.001–1.084)	0.045		
A	1.013 (0.905–1.135)	0.818			1.004 (0.895–1.126)	0.945		

Characteristics	Male: Univariate		Male: Multivariate		Female: Univariate		Female: Multivariate	
ALT	1.051 (1.018–1.086)	0.002	1.029 (0.993–1.066)	0.114	1.067 (1.017–1.120)	0.008		
AST	1.063 (1.012–1.118)	0.015			1.019 (0.998–1.040)	0.078		
Γ-GT	1.005 (0.998–1.012)	0.159			1.071 (1.024–1.121)	0.003		
TB	0.988 (0.945–1.033)	0.604			1.001 (0.945–1.060)	0.977		
DB	0.991 (0.870–1.129)	0.894			1.008 (0.836–1.215)	0.935		
IDB	0.990 (0.937–1.045)	0.704			1.001 (0.932–1.076)	0.971		
APRI	8.729 (3.019–25.238)	<0.001	4.552 (1.120–18.500)	0.034	1.165 (0.114–11.929)	0.898		
GPR	1.913 (1.291–2.833)	0.001			15.940 (0.238–1066)	0.197		
FIB4	0.994 (0.709–1.394)	0.972			0.682 (0.252–1.846)	0.451		
NLR	1.044 (0.748–1.457)	0.799			0.989 (0.641–1.525)	0.958		
PLR	0.997 (0.989–1.005)	0.479			1.010 (1.001–1.019)	0.035	1.013 (1.004–1.023)	0.007
RBC	1.525 (0.722–3.224)	0.269			0.921 (0.299–2.834)	0.886		

Characteristics	Male: Univariate		Male: Multivariate		Female: Univariate		Female: Multivariate	
HB	0.978 (0.955–1.003)	0.085			0.998 (0.962–1.035)	0.910		
WBC	0.978 (0.789–1.213)	0.841			0.921 (0.676–1.255)	0.603		
N	0.948 (0.726–1.238)	0.695			0.954 (0.670–1.359)	0.794		
L	0.927 (0.559–1.536)	0.768			0.805 (0.333–1.948)	0.630		
PLT	0.998 (0.992–1.004)	0.518			1.007 (0.999–1.014)	0.099		
AFP	1.009 (1.002–1.017)	0.012	1.018 (1.009–1.026)	<0.001	0.998 (0.971–1.025)	0.884		

HB was significantly associated with phase transition in the multivariate model, but this association was attenuated after adjustment for sex alone (HR 0.985, P=0.139), suggesting possible confounding by sex.

B. Antiviral therapy

Characteristics	Male: Univariate		Male: Multivariate		Female: Univariate		Female: Multivariate	
	HR (95% CI)	P	HR (95% CI)	P	HR (95% CI)	P	HR (95% CI)	P
Group		0.400		0.640		<0.001		0.0005
GZIC	Reference		Reference		Reference		Reference	
IC	0.79 (0.46–1.38)		0.84 (0.40–1.76)		0.25 (0.14–0.45)		0.32 (0.17–0.61)	
family		0.720				0.608		
NO	Reference				Reference			
YES	1.21 (0.43–3.38)				1.24 (0.55–2.77)			
age	1.024 (0.995–1.055)	0.107			1.015 (0.987–1.045)	0.301		
A	0.954 (0.843–1.080)	0.459			0.963 (0.913–1.015)	0.161		
ALT	0.982 (0.952–1.013)	0.259			1.019 (0.982–1.057)	0.326		
AST	1.019 (0.960–1.082)	0.540			1.038 (1.014–1.062)	0.002	—	—
Γ-GT	1.001 (0.992–1.010)	0.854			1.018 (0.971–1.067)	0.463		

Characteristics	Male: Univariate		Male: Multivariate		Female: Univariate		Female: Multivariate	
TB	1.022 (0.978–1.067)	0.333			0.968 (0.915–1.025)	0.262		
DB	0.998 (0.859–1.160)	0.979			0.966 (0.839–1.112)	0.629		
IDB	1.022 (0.969–1.077)	0.426			0.946 (0.871–1.028)	0.191		
APRI	5.224 (1.527–17.874)	0.008	10.273 (2.20–47.985)	0.003	7.451 (2.662–20.858)	<0.001	3.696 (1.234–11.071)	0.020
GPR	1.696 (1.027–2.803)	0.039			17.698 (0.997–314.1)	0.050		
FIB4	1.235 (1.010–1.510)	0.040			1.151 (0.701–1.891)	0.578		
NLR	1.212 (0.857–1.715)	0.276			1.111 (0.828–1.491)	0.483		
PLR	0.998 (0.989–1.006)	0.562			1.001 (0.992–1.009)	0.906		
RBC	0.427 (0.193–0.942)	0.035	0.388 (0.16–0.940)	0.036	0.852 (0.389–1.867)	0.689		
HB	0.990 (0.963–1.018)	0.478			1.027 (0.997–1.058)	0.079		
WBC	0.903 (0.710–1.149)	0.406			0.999 (0.807–1.236)	0.991		
N	0.968 (0.730–1.284)	0.824			1.030 (0.809–1.311)	0.813		

Characteristics	Male: Univariate		Male: Multivariate		Female: Univariate		Female: Multivariate	
L	0.624 (0.331–1.177)	0.145			0.793 (0.423–1.485)	0.468		
PLT	0.994 (0.988–1.001)	0.095			0.997 (0.991–1.002)	0.211		
AFP	1.013 (1.006–1.021)	<0.001	1.019 (1.010–1.028)	<0.001	0.998 (0.971–1.025)	0.872		

C. End-stage liver disease

Due to the small number of events (male: 6; female: 4), formal Cox regression was not performed. Only descriptive data are presented.

Outcome	Male IC	Male GZIC	Female IC	Female GZIC
End-stage liver disease, n (%)	1 (0.9)	5 (6.2)	3 (2.0)	1 (2.3)

HR, hazard ratio; CI, confidence interval. HRs compare GZIC vs IC within each sex. Variables with P<0.05 in univariate analysis were entered into the multivariate model.

Due to collinearity among fibrosis markers (APRI, GPR, FIB4), only APRI was retained in the male phase transition and antiviral therapy models. For the male antiviral therapy model, state was forced into the model as the primary exposure variable. For the female phase transition model, only state and PLR were retained because the limited number of events (n=11) precluded a larger model. The end-stage liver disease analysis should be interpreted as exploratory.
